# Supplementary material for: Differences in Stakeholders’ Perception of the Impact of COVID-19 on Clinical Care and Decision-Making
Source: Cancers (Basel). 2022 Sep 2;14(17):4317. doi: 10.3390/cancers14174317 (PMC9454870; doi:10.3390/cancers14174317)
Supplement: Supplementary file 1 [file cancers-14-04317-s001.zip › cancers-1874515-supplementary.pdf]

Supplementary Materials

Differences in Stakeholders' Perception of the Impact of COVID-19 on Clinical Care and Decision-Making

Table S1. Significant differences of decisional uncertainty between stakeholder groups. Multivariate comparison was done using Tukey-HSD test.

Stakeholder in Entity Group	Stakeholder in Entity Group	Mean Differences	Std.-Div.	Sig.	95% Confidence Interval	
					Lower Level	Upper Level
Oncology Physician	Oncology Nurses	−0.589 *	0.097	0.000	−0.87	−0.31
	Oncology Patient	0.349 *	0.088	0.001	0.10	0.60
	Psychiatry Physician	−0.538 *	0.119	0.000	−0.88	−0.20
	Psychiatry Nurses	−1.167 *	0.115	0.000	−1.49	−0.84
	Psychiatry Patient	−0.417 *	0.110	0.002	−0.73	−0.10
Oncology Nurses	Oncology Physician	0.589 *	0.097	0.000	0.31	0.87
	Oncology Patient	0.938 *	0.085	0.000	0.70	1.18
	Psychiatry Physician	0.051	0.116	0.998	−0.28	0.38
	Psychiatry Nurses	−0.578 *	0.112	0.000	−0.90	−0.26
	Psychiatry Patient	0.172	0.107	0.588	−0.13	0.48
Oncology Patient	Oncology Physician	−0.349 *	0.088	0.001	−0.60	−0.10
	Oncology Nurses	−0.938 *	0.085	0.000	−1.18	−0.70
	Psychiatry Physician	−0.887 *	0.109	0.000	−1.20	−0.58
	Psychiatry Nurses	−1.516 *	0.105	0.000	−1.81	−1.22
	Psychiatry Patient	−0.765 *	0.099	0.000	−1.05	−0.48
Psychiatry Physician	Oncology Physician	.538 *	0.119	0.000	0.20	0.88
	Oncology Nurses	−0.051	0.116	0.998	−0.38	0.28
	Oncology Patient	0.887 *	0.109	0.000	0.58	1.20
	Psychiatry Nurses	−0.630 *	0.131	0.000	−1.00	−0.26
	Psychiatry Patient	0.121	0.127	0.932	−0.24	0.48
Psychiatry Nurses	Oncology Physician	1.167 *	0.115	0.000	0.84	1.49
	Oncology Nurses	0.578 *	0.112	0.000	0.26	0.90
	Oncology Patient	1.516 *	0.105	0.000	1.22	1.81
	Psychiatry Physician	0.630 *	0.131	0.000	0.26	1.00
	Psychiatry Patient	0.751 *	0.123	0.000	0.40	1.10
Psychiatry Patient	Oncology Physician	0.417 *	0.110	0.002	0.10	0.73
	Oncology Nurses	−0.172	0.107	0.588	−0.48	0.13
	Oncology Patient	0.765 *	0.099	0.000	0.48	1.05
	Psychiatry Physician	−0.121	0.127	0.932	−0.48	0.24
	Psychiatry Nurses	−0.751 *	0.123	0.000	−1.10	−0.40

*. Significant differences $p < 0.05$.
